# Integration of Transcriptomics and Metabolomics Reveals the Antitumor Mechanism Underlying Tadalafil in Colorectal Cancer

**DOI:** 10.3389/fphar.2022.793499

**Published:** 2022-05-27

**Authors:** Pan Zhao, Yao Shen, Mengyang Li, Hanjun Dan, Zhiming Zhao, Jian Zhang

**Affiliations:** ^1^ The State Key Laboratory of Cancer Biology, Department of Biochemistry and Molecular Biology, The Fourth Military Medical University, Xi’an, China; ^2^ The Faculty of Hepatopancreatobiliary Surgery, The First Medical Center, Chinese People’s Liberation Army General Hospital, Beijing, China

**Keywords:** tadalafil, colorectal cancer, transcriptomics, metabolomics, alanine, aspartate, and glutamate metabolism

## Abstract

The potential role of tadalafil, a PDE5 inhibitor, in anticancer activity and prolonged survival has been proposed. However, the systematic effects of tadalafil in colorectal cancer were not fully understood. In this study, we assessed the anti-tumor activity of tadalafil in human colorectal cancer cells. A systematic perspective of the tadalafil-induced anti-tumor mechanism was provided by the integration of transcriptomics and metabolomics. We found that differentially expressed genes (DEGs) were mainly involved in microRNAs in cancer, purine metabolism, glycosphingolipid biosynthesis, arginine biosynthesis, and amino acid metabolism. Amino acid metabolism, especially alanine, aspartate, and glutamate metabolism was the most of the differentially accumulated metabolites (DAMs) through the analysis of metabolomics. The conjoint analysis of DEGs and DAMs presented that they were also mainly involved in alanine, aspartate, and glutamate metabolism. Amino acid metabolism-related genes, *GPT, GGT5, and TAT*, were significantly decreased after tadalafil treatment. In particular, the disturbance of alanine, aspartate, and glutamate metabolism may be the explanation for the major mechanism resulting from tadalafil anti-tumor activity.

## Introduction

Tadalafil, a phosphodiesterase type5 (PDE5) inhibitor, was developed as a treatment for erectile dysfunction (Padma-Nathan et al., 2001), pulmonary arterial hypertension, and benign prostatic hyperplasia (Roehrborn et al., 2010). A range of PDE5 inhibitors, such as tadalafil, sildenafil, vardenafil, and exisulind, were all proved to have anti-tumor properties. Exisulind, a drug of PDE5 inhibition, was first shown to have anti-tumor activity by inducing cancer cell apoptosis in the SW480 colon tumor cell line in 2000 (Thompson et al., 2000). Subsequently, sildenafil and vardenafil were suggested to cause caspase-dependent apoptosis in patient-derived B-cell chronic lymphocytic leukemia cells (Sarfati et al., 2003). In recent years, there were significantly increasing research studies focused on the anti-tumor properties of PDE5 inhibitors both in academic and clinic, especially when drug repurposing has become a very effective strategy for both cancer prevention and therapy. Tadalafil is an important member of the PDE5 inhibitor family, and there were emerging evidences which have confirmed its anti-tumor effects (Serafini et al., 2006; Booth et al., 2014a; Booth et al., 2014b; Roberts et al., 2014; Califano et al., 2015; Sponziello et al., 2015; Wang et al., 2015; Weed et al., 2015; De Rose et al., 2016; Tuttle et al., 2016; Booth et al., 2017; Hassel et al., 2017; Califano et al., 2018). The different mechanisms’ relation to the anti-tumor effects of tadalafil existed, including restoring immunosuppression (Serafini et al., 2006), increasing chemotherapeutic sensitization (Wang et al., 2015), reversing hypoxia-induced resistance (Ammirante et al., 2014), and inhibiting proliferation and migration (De Rose et al., 2016). Recently, two double-blind, randomized, placebo-controlled clinical trials suggested that the PDE5 inhibitors have anti-tumor effects on colorectal cancer (CRC) in a national cohort of patients. In particular, they found that 10-mg dosage of tadalafil could reduce the damage of colorectal cancer by 1.7%, and PDE5 inhibitors could be a potential adjuvant drug to improve prognosis for patients with CRC (Huang et al., 2020; Sutton et al., 2020). There has also been evidence which demonstrated that tadalafil suppressed CRC at a cellular level (Serafini et al., 2006); however, the pharmacological mechanism of tadalafil in colorectal cancer was not fully understood yet.

The incidence of CRC is increasing worldwide, which has become a significant global health burden and is the second and third most commonly diagnosed cancer worldwide in women and men, respectively, (Siegel et al., 2019). Over the past decades, the prognosis of CRC has improved, but according to recent data which is with a trend of slowing down (Brenner et al., 2014; Siegel et al., 2019). Surgery still is the first choice of therapy for patients with CRC (Behrenbruch et al., 2018). The discovery of new therapeutic strategies in CRC treatment has become an urgent need and also attracted great research interest (Nappi et al., 2018). Emerging evidence has indicated that cancer is mainly a metabolic disease involving metabolism reprogramming such as Warburg effect (Seyfried et al., 2014). The analysis of metabolomics has suggested multiple altered metabolites in bio-fluids and tissues of CRC patients, including cellular respiration/carbohydrate, amino acid, lipid, nucleotide, and other significant metabolite perturbations (Goedert et al., 2014; Farshidfar et al., 2016; Uchiyama et al., 2017). So, we focus the therapeutic strategies for CRC on metabolism reprogramming.

At present, transcriptomics and metabolomics were used to evaluate the effect of tadalafil on CRC. In order to further investigate the potential transcript alterations, metabolic alterations, and molecular mechanisms, we performed systemic correlation networks of similarities in the two omics data. This study aimed to identify the key metabolites in tadalafil-treated CRC, which hoping to provide a new and systematic insight into tadalafil on CRC.

## Materials and Methods

### Cell Cultures and Treatments

The human colorectal cancer cell lines HCT116, HT29, and SW620 were purchased from the American Type Culture Collection (ATCC, Rockville, MD). The cells were cultured with McCoy’s 5A medium supplemented with 1% penicillin/streptomycin and 10% fetal calf serum. An atmosphere of 5% CO_2_ at 37°C was used to culture cells. The cells (5 × 10^6^ cells) were seeded in a 10-cm plate and exposed to 10 μM of tadalafil (HY-90009A, MedChemExpress, United States) for 24 h when cells had reached 50–60% confluence.

### Cell Viability Assay

The three cell lines were seeded into 96-well plates (Conning, United States) at a density of 1.0 × 10^4^ cells/ml, cultured at 37°C for 12 h, and then treated with tadalafil at different concentrations for 24 h. Each well of the plate was added with 10 μl of CCK-8 solution and incubated for 2.5 h. The absorbance was measured at 450 nm using a microplate reader.

### RNA Preparation, cDNA Synthesis, and Sequencing

After HCT116 cells were treated with 10 μM tadalafil for 24 h, the total RNA was extracted from cells using the RNAiso Plus (TRIzol) reagent according to the manuscript’s protocol. To meet the requirements for the RNA sequence, 1% agarose electrophoresis was employed to test purity; meanwhile, a Qubit^®^ 2.0 fluorometer (Life Technologies, CA, United States) and 2100 RNA Nano 6000 assay kit (Agilent Technologies, CA, United States) were used to detect content and integrity of the RNA, respectively. Then, poly-T oligo-attached magnetic beads were used to purify mRNA from total RNA. Next, the first- and second-strand cDNA was synthesized using M-MuLV reverse transcriptase and DNA polymerase I and RNase H, respectively. Then, size-selected and adaptor-ligated cDNA was performed with USER enzyme (NEB, United States) at 37°C for 15 min followed by 5 min at 95°C before PCR. The Illumina HiSeqTM2000/MiSeq platform (Cipher Gene LLC, Beijing, China) was applied to carry out RNA sequencing.

### Liquid Chromatography–Tandem Mass Spectrometry (LC-MS/MS)

HCT116 cells, with or without 10 μM tadalafil treatment for 24 h, were collected using centrifugation at 12,000 rpm at 4°C for 10 min after washing with PBS three times, and then the cell pellets were quickly stored at −80°C until subsequent metabolomics assays. Briefly, analyses were performed using an UHPLC (1290 Infinity LC, Agilent Technologies) coupled to a quadrupole time-of-flight (AB Sciex TripleTOF 6600) in Shanghai Applied Protein Technology Co., Ltd. For HILIC separation, samples were analyzed using a 2.1 mm × 100 mm ACQUIY UPLC BEH 1.7-μm column (Waters, Ireland). For RPLC separation, a 2.1 mm × 100 mm ACQUIY UPLC HSS T3 1.8-μm column (Waters, Ireland) was used.

The raw MS data (wiff.scan files) were converted to MzXML files using ProteoWizard MSConvert before importing into freely available XCMS software. Collection of Algorithms of MEtabolite pRofile Annotation (CAMERA) was used for annotation of isotopes and adducts. In the extracted ion features, only the variables having more than 50% of the non-zero measurement values in at least one group were included. Compound identification of metabolites was performed by comparing accuracy of the m/z value (< 25 ppm), and MS/MS spectra with an in-house database were established with available authentic standards.

### Data Processing

After being normalized to total peak intensity, an R package was employed to analyze the processed data. Multivariate data analysis, including Pareto-scaled principal component analysis (PCA) and orthogonal partial least-squares discriminant analysis (OPLS-DA), was performed to evaluate the differences between groups. For samples with biological duplications, they are analyzed using DESeq and the normalization method of quantile.

Gene Ontology (GO) annotations for DEGs were implemented by an R package plug-in clusterProfiler. The Kyoto Encyclopedia of Genes and Genomes (KEGG) database was employed to analyze the higher advanced features, practical application of genomic information, and the large-scale datasets produced via transcriptome and metabolome. The clusterProfiler R package was also used to detect the statistical enrichment of differentially expression genes in KEGG pathways.

### Joint Enrichment Analyses on Transcript and Metabolite Patterns

All DEGs and DAMs were referred and mapped to pathways according to the online KEGG (http://www.kegg.jp/). Enrichment analysis was also performed. R version 3.5.1 was used to combine the KEGG annotation and enrichment result of the two omics. The Venn diagram and bar plot were drawn.

Differentially abundant genes and metabolites were log2 scaled (TMT/iTRAQ) or Z-score scaled (label-free) and concatenated into one matrix. Then, the correlation coefficient among all the molecules in the matrix was calculated with Pearson’s algorithm in R version 3.5.1.

### Real-Time Quantitative PCR

The total RNA was extracted from culture cells by TRIzol reagent, and PrimeScriptTM II 1st Strand cDNA Synthesis Kit (Vazyme Biotech Co., Ltd) was used to synthesize cDNA with mRNA-specific primers. A Bio-Rad C1000 Real-Time PCR machine was used for the real-time quantitative PCR. The 2^−ΔΔCT^ method was used to determine the relative expression of targeted genes, and all reactions were repeated three times. The sequences of primers are listed in Supplementary Table S1.

### Statistical Analysis

The DESeq2 R package (1.16.1) was used to perform the differential expression analysis of two groups (three biological replicates per group). Statistical routines for determining differential expression in digital gene expression data were provided by DESeq2, using a model based on the negative binomial distribution.

To control the false discovery rate, the Benjamin and Hochberg’s approach was used to adjust the resulting *p*-values. Genes with an adjusted *p*-value < 0.05 found by DESeq2 were considered differentially expressed.

After being normalized to total peak intensity, the processed data were analyzed by R package (ropls), in which it was subjected to multivariate data analysis, including Pareto-scaled principal component analysis (PCA) and orthogonal partial least-squares discriminant analysis (OPLS-DA). The seven-fold cross-validation and response permutation testing was used to evaluate the robustness of the model. The variable importance in the projection (VIP) value of each variable in the OPLS-DA model was calculated to indicate its contribution to the classification. Metabolites with the VIP value > 1 was further applied to Student’s t-test at a univariate level to measure the significance of each metabolite. The *p*-values < 0.05 were considered statistically significant.

## Results

### Anti-Tumor Effect of Tadalafil on Human Colon Cancer Cell Lines

**FIGURE 1:**
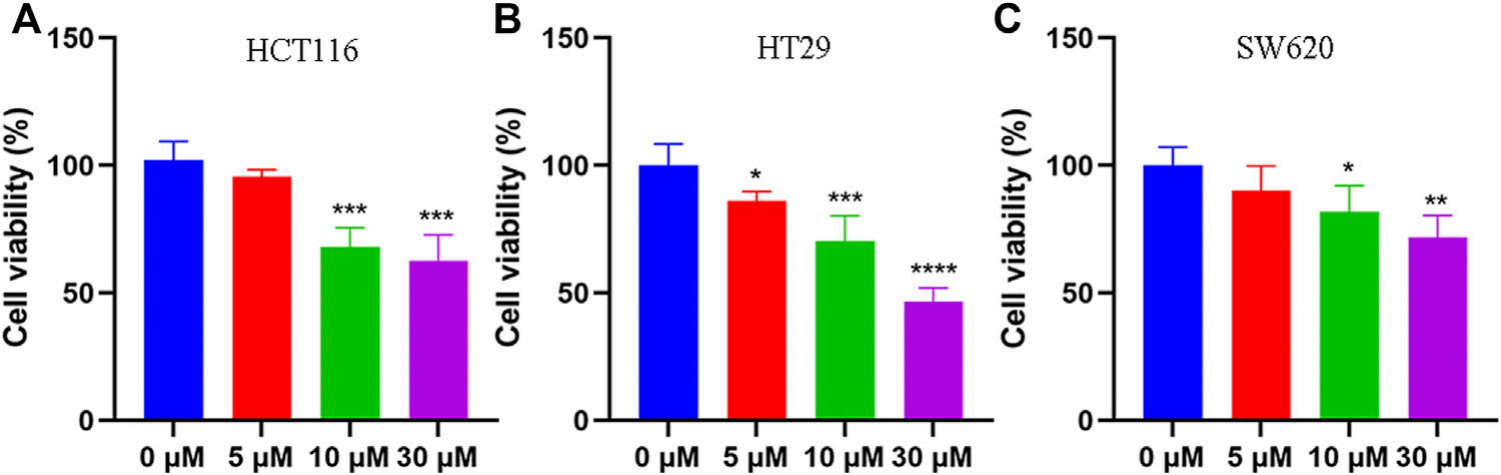
Anti-tumor effect of tadalafil on human colon cancer cell lines. **(A)** HCT116 cells. **(B)** HT29 cells. **(C)** SW620 cells. Data are expressed as mean ± SD, *n* = 5 (*p-value < 0.05, ** *p*-value < 0.01, and *** *p*-value < 0.001).

To elucidate the anti-tumor effect of tadalafil on human colon cancer cell lines, we used MTT assay and colony formation to analyze the growth and proliferation of colorectal cancer cell HCT116 after tadalafil treatment for 24 h. Our data showed that tadalafil could inhibit the growth and proliferation of HCT116 cells (data not shown). In addition, the cell viability of human colon cancer cell lines, HCT116, HT29, and SW620, was analyzed by CCK-8 assay; the data suggested that different concentrations of tadalafil treatment for 24 h had cytotoxicity and inhibited cell proliferation (Figure 1).

### Transcriptomics Analysis Results of Tadalafil-Treated HCT116 Colorectal Cancer Cell

**FIGURE 2:**
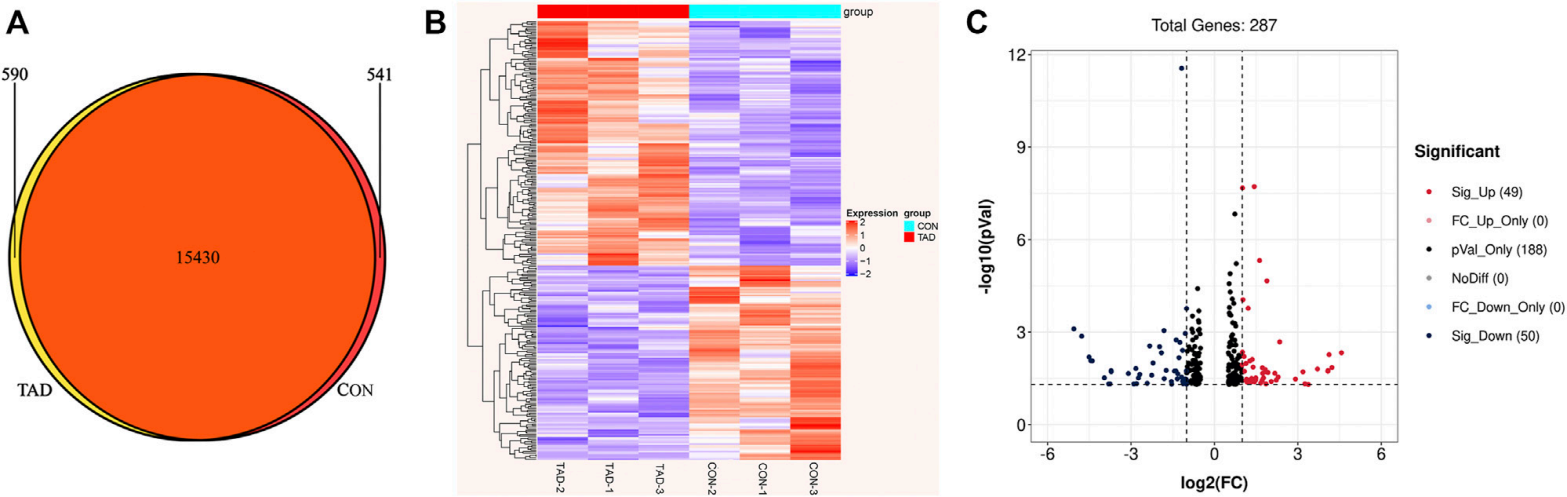
Transcriptional differences between colorectal cancer HCT116 cells treated with tadalafil (TAD) and without tadalafil (CON). **(A)** Venn diagram of transcriptome profiles in TAD and CON groups. Gene expression analysis revealed that 15,430 genes were co-expressed in TAD and CON groups, while 590 and 541 genes were specifically expressed in TAD and CON groups, respectively. **(B)** Cluster heatmap of differentially expressed genes (DEGs) induced by tadalafil in HCT116 cells; the green to red color scale indicates the value of log2 (FPKM) after scale number. **(C)** Volcano plot of DEGs; the green indicates downregulated DEGs, and red indicates upregulated DEGs. *p*-value < 0.05 and FC ≥ 2.

To identify the tadalafil-related deferentially expressed genes, we analyzed the transcriptome profiles of HCT116 cells with or without tadalafil treatment after RNA sequencing. The Venn diagram of transcriptome profiles in tadalafil (TAD) and control (CON) groups was shown. Gene expression analysis revealed that 15,430 genes were co-expressed in TAD and CON groups, while 590 and 541 genes were specifically expressed in TAD and CON groups, respectively (Figure 2A). After filtering out adapter sequences and the terrible reads, 287 DEGs (160 upregulated genes and 127 downregulated genes) were identified (Figures 2B,C). The criteria of *p*-value < 0.05 and FDR ≥2 statistical analysis were used.

### GO Enrichment and KEGG Analysis of Metabolites

**FIGURE 3:**
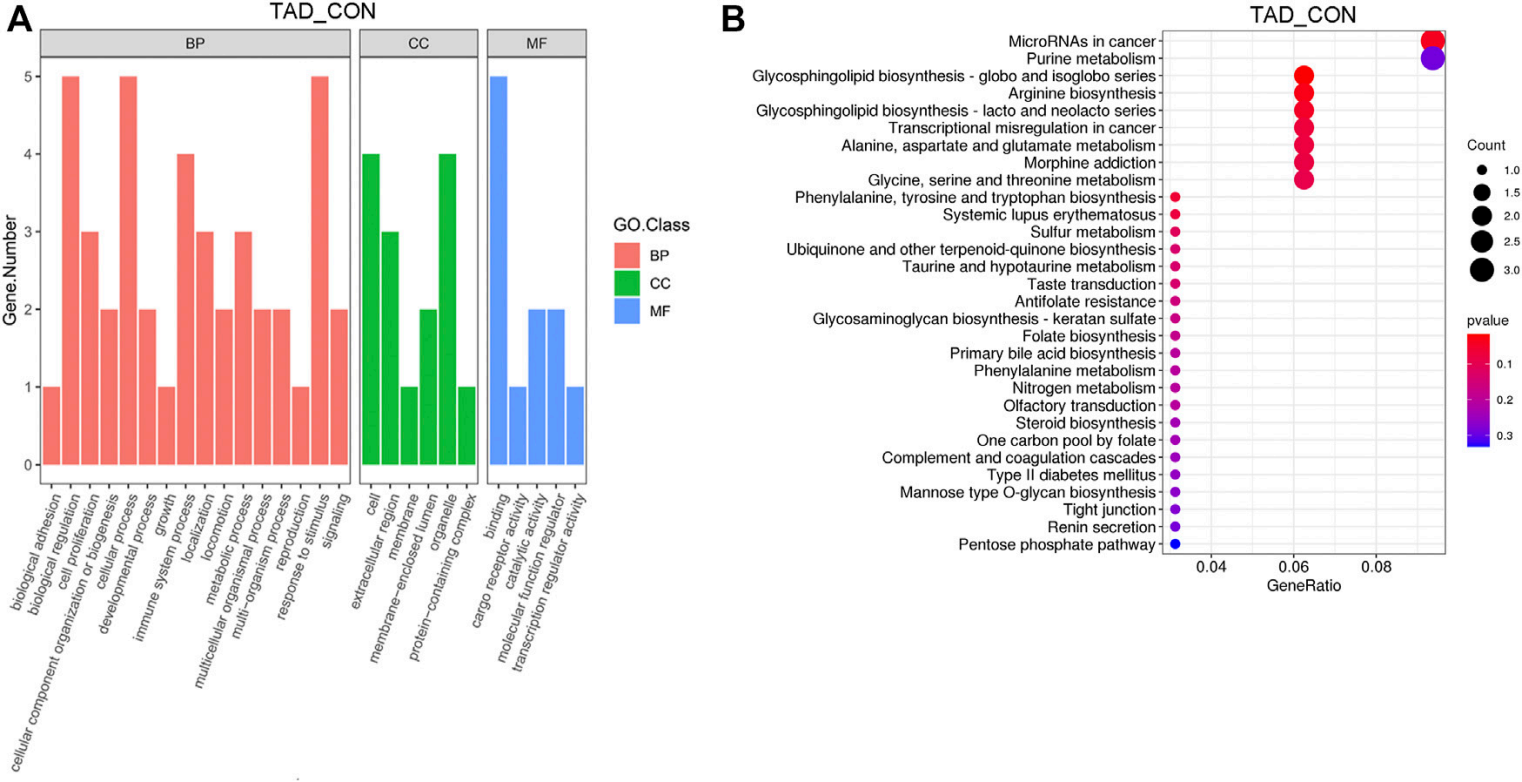
Functional enrichment analysis of DEGs. **(A)** Functional enrichment clustering analysis of DEGs was performed by top GO terms in biological process, cellular component, and molecular function. **(B)** Bubble chart of the KEGG pathways (top 30) that DEGs significantly involved in. The rich factor is defined as the ratio of the number of DEGs to the total number of genes enriched in a specific category. The count of DEGs was roughly represented by the size of circles. Log10-adjusted *p*-value was indicated by the color saturation from green to red. GO, Gene Ontology; BP, biological processes; CC, cellular components; MF, molecular functions; KEGG, Kyoto Encyclopedia of Genes and Genomes; DEGs, differentially expressed genes. *p*-value < 0.05.

After removing genes with low expression, main GO functional characterization was conducted, which included biological process (BP), cellular component (CC), and molecular function (MF). The criteria of *p* < 0.05 statistical analysis were used. According to the expression level of the DEGs and *p*-value, top 27 unique GO terms were assigned to all DEGs. The present results showed that the most prominent GO terms were response to toxic substance, extracellular space, and chemoattractant activity in TAD groups compared with CON groups (Figure 3A). To further understand the enriched pathways of these DEGs which are significantly differentially expressed in TAD groups compared with CON groups, we carried out the KEGG pathway analysis and found that the most of DEGs involved in metabolic pathways, in which microRNA in cancer was the most significantly enriched pathway (Figure 3B). In addition, purine metabolism, glycosphingolipid biosynthesis, arginine biosynthesis, and multi-amino acid metabolism are all enriched pathways using KEGG analysis (Figure 3B).

### Tadalafil-Induced Metabolite Profile Alterations

**FIGURE 4:**
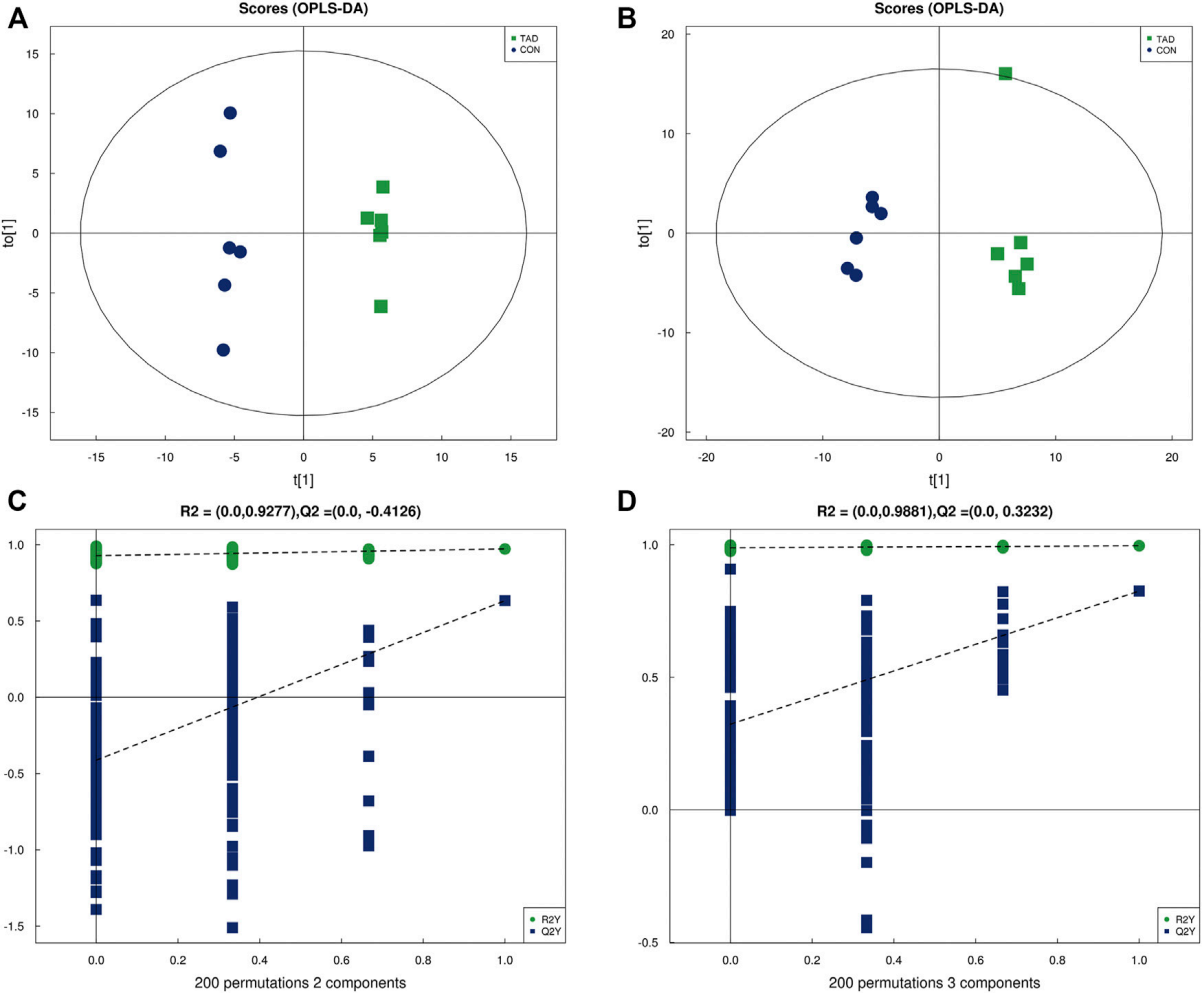
OPLS-DA analysis of alterations in the metabolite profile. **(A)** OPLS-DA score plot derived from metabolite ions acquired using the ESI+. **(B)** OPLS-DA score plot derived from metabolite ions acquired using the ESI-. **(C,D)** Permutation test for OPLS-DA in positive and negative ion mode. OPLS-DA, orthogonal partial least-squares discriminant analysis; ESI+, electrospray ionization positive ion mode; ESI-, electrospray ionization negative ion mode.

OPLS-DA was employed to systematically analyze all DAMs. The difference in observations was directly proportional to the distance of the points in the OPLS-DA score graph. The results showed that the significant changes of the main biological components were along with tadalafil treatment (Figures 4A,B). An obvious separation between two groups was presented by the score plots of OPLS-DA. The OPLS-DA model exhibited the R2Y and Q2 values as 0.972 and 0.633 in the positive ion OPLS-DA model. The values of R2Y and Q2 were 0.996 and 0.825 in the negative ion OPLS-DA model. These data suggested highly significant differences of metabolite profiles of HCT116 cells treated with or without tadalafil (Figures 4A,B). The reliability of OPLS-DA was evaluated by the permutation test. The horizontal axis represents the retention, and the coordinate axis represents the value of R2Y or Q2 in the model verification permutation test graph. The stability and reliability of the model were presented by the model, in which all Q2 points were lower than the original blue Q2 points (Figures 4C,D).

**FIGURE 5:**
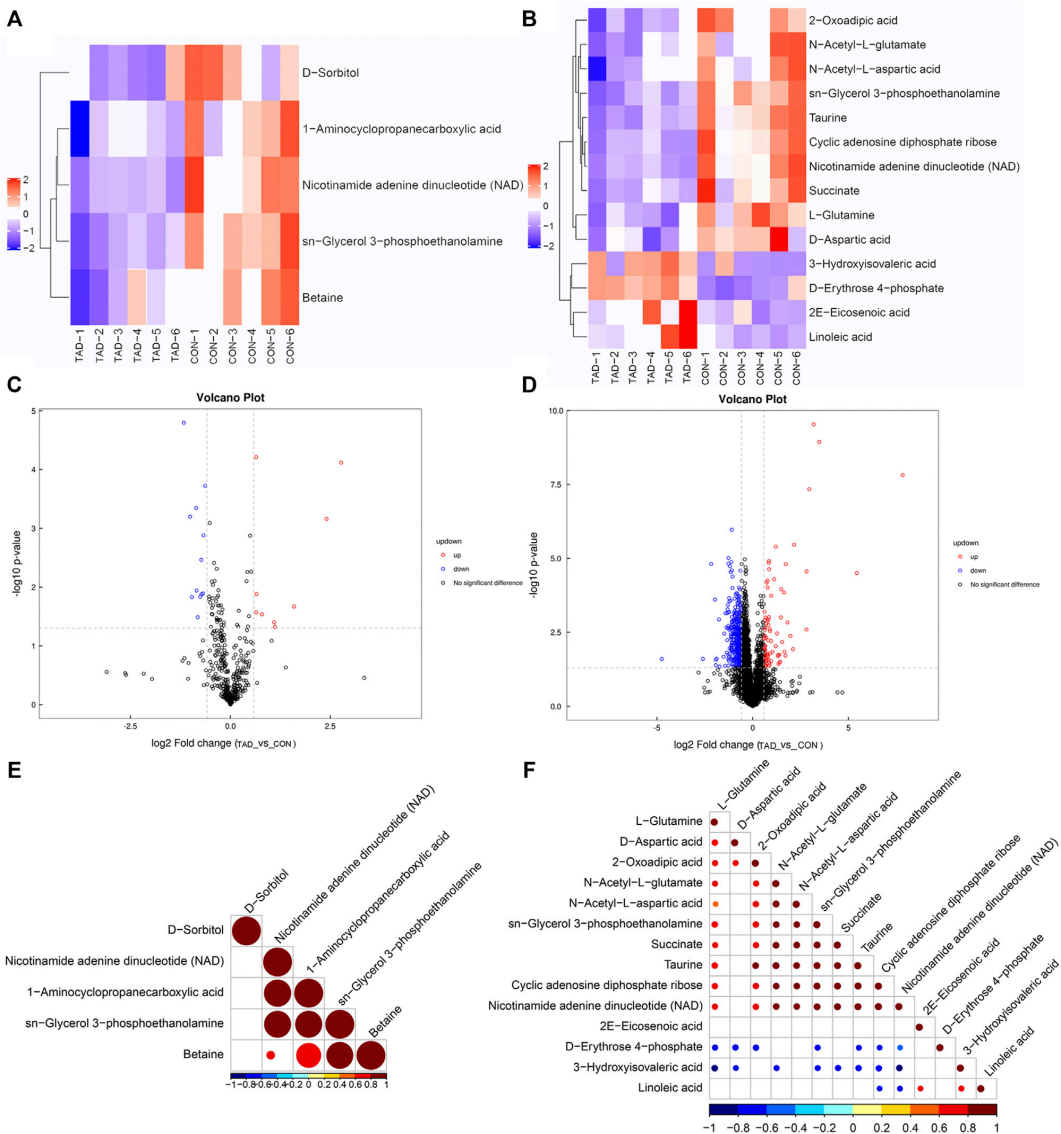
Metabolic differences between colorectal cancer HCT116 cells treated with tadalafil groups (TAD) and without groups (CON). **(A,B)** Cluster heatmap of DAMs in ESI+ and ESI-. The value of DAMs expression after scale number was indicated by the blue to red color scale. **(C,D)** Volcano plots of DAMs in ESI+ and ESI−. Red indicates upregulated accumulated metabolites (*p*-value <0.05, FC > 1.5), and blue indicates downregulated accumulated metabolites (*p*-value <0.05, FC < 0.67). Black indicates no significant difference of metabolites. **(E,F)** Pearson correlation analysis of the DAMs based on *p* < 0.05 and VIP >1 in ESI+ and ESI−. The value of the Pearson correlation coefficient was represented by the color label. ESI+, electrospray ionization positive ion mode; ESI−, electrospray ionization negative ion mode; DAM, differentially accumulated metabolites.

According to the *p*-value and fold changes of DAMs (FC > 1.5 and *p*-value <0.05 of DAMs indicated upregulation; FC < 0.67 and *p* value < 0.05 of DAMs indicated downregulation), the heatmaps (Figures 5A,B) and volcano plots (Figures 5C,D) were plotted to distinguish the difference in positive or negative correlation of DAMs. The criteria of either *p* < 0.05 at univariate statistics or VIP > 1 at multivariate statistical analysis were used. Five metabolites were identified under positive ion mode, and 14 metabolites were identified under negative ion mode. Moreover, Pearson’s correlation coefficients between DAMs and correlation matrixes were calculated and visualized as heatmaps (Figures 5E,F).

**FIGURE 6:**
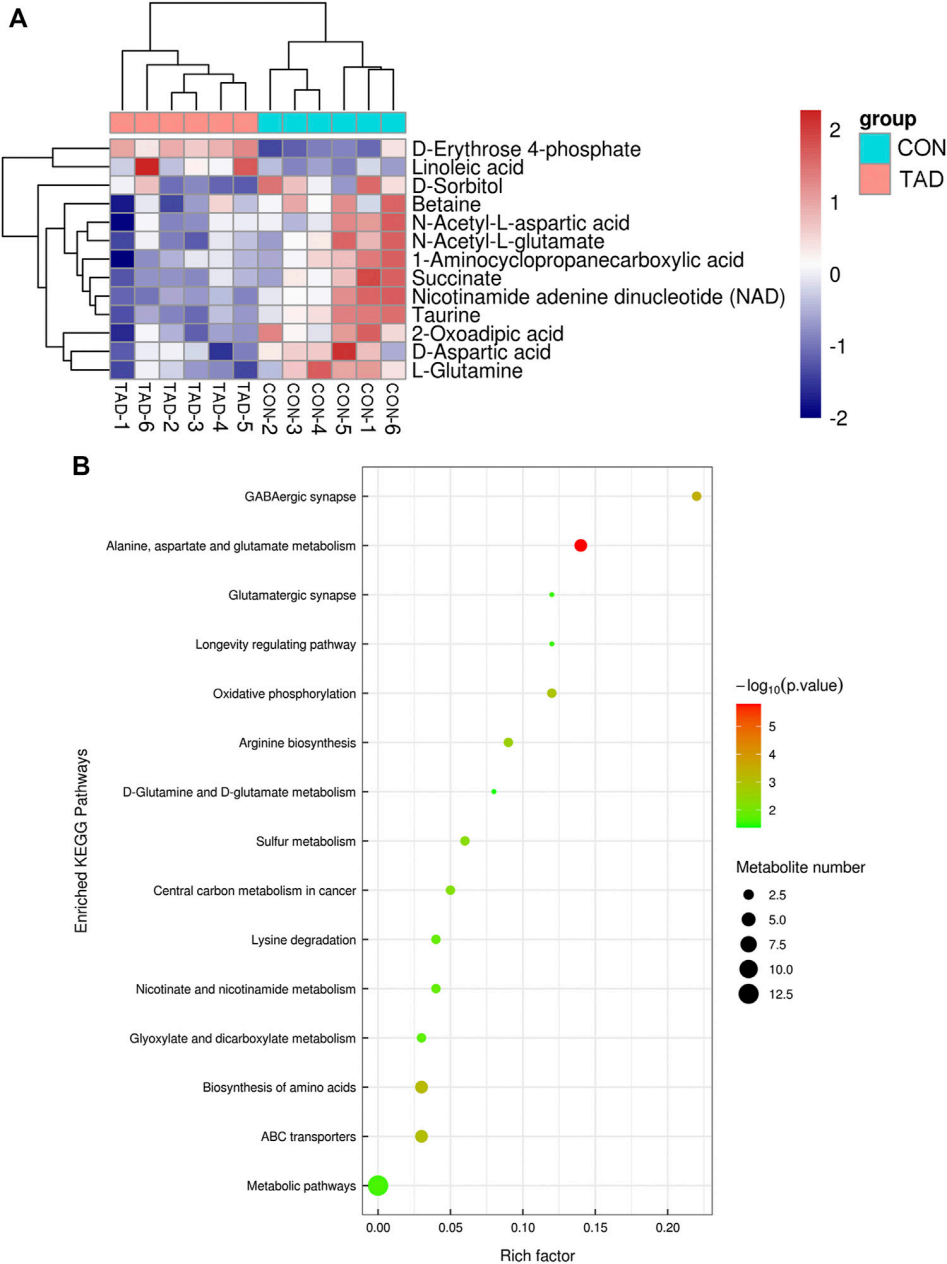
Functional enrichment analysis of DAMs. **(A)** Heatmap of KEGG pathways. **(B)** Bubble chart of the KEGG pathways (top 15) that DAMs significantly involved in. The rich factor is defined as the ratio of the number of DEGs to the total number of genes enriched in a specific category. The count of DEGs was roughly represented by the size of circles. Log10-adjusted *p* value was indicated by the color saturation from green to red.

To determine whether the differential metabolites were involved in the biological pathways, KEGG pathways analysis was used to annotate all differential metabolites. The metabolite heatmaps and bubble chart are shown in Figures 6A,B. The majority of metabolites were emerged in alanine, aspartate, and glutamate metabolism pathways (Figure 6B).

### Integrated Analysis of Transcript and Metabolite Profiles

**FIGURE 7:**
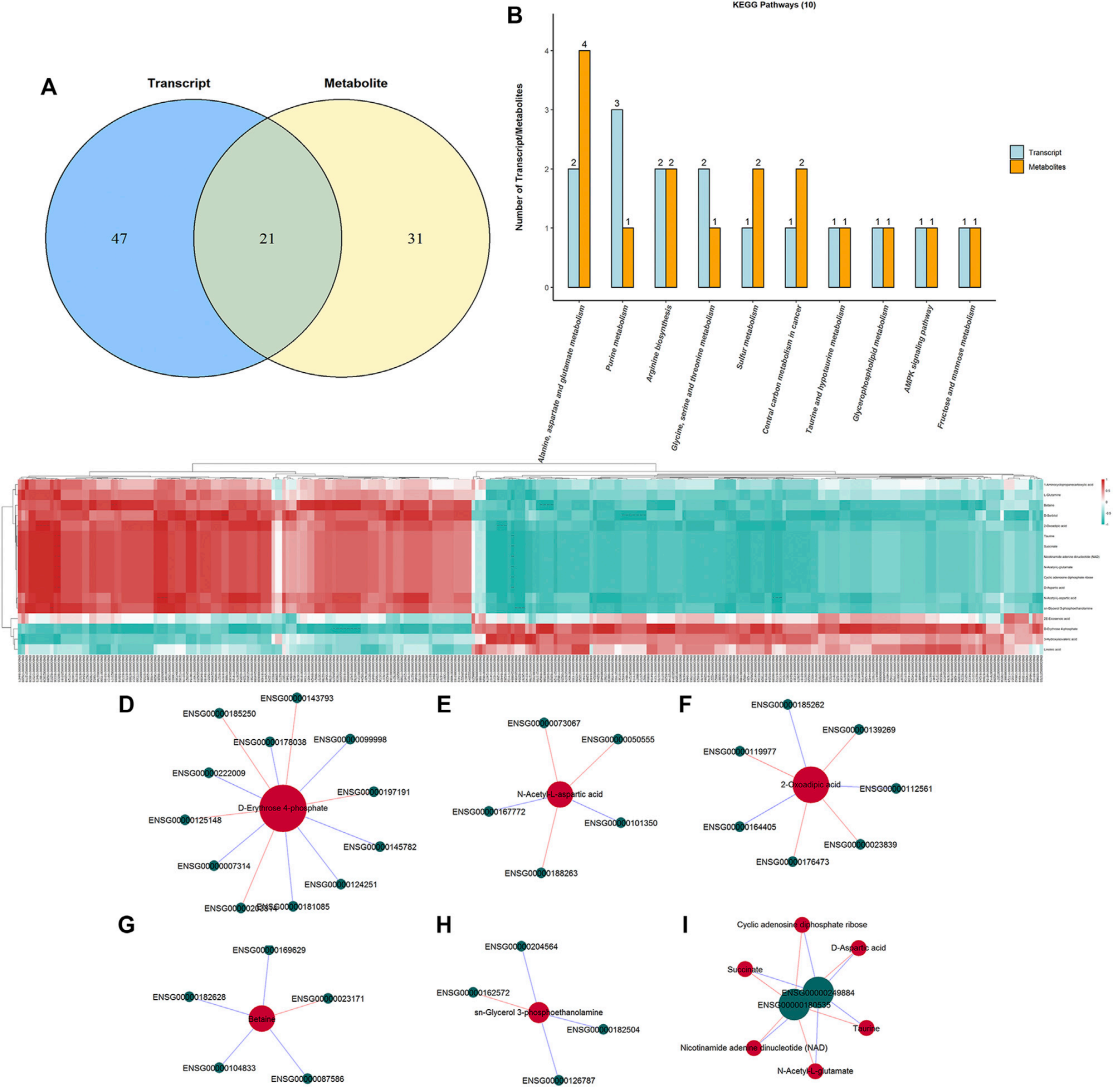
Integrated enrichment analysis of transcript and metabolite profiles. **(A)** KEGG pathways that DEGs and DAMs both enriched in were performed using a Venn plot. **(B)** Histogram of the KEGG pathways (top 10) that DEGs and DAMs both enriched in. **(C)** Heatmap of the correlation coefficient matrix between DEGs and DAMs analyzed by the Pearson correlation. Red indicates the positive correlation, and green indicates negative correlation. The value of the Pearson correlation coefficient was represented by the color label. The correlation coefficient R is shown in color. R > 0 indicates positive correlation, represented by red; R < 0 indicates negative correlation, represented by green; and darker color indicates stronger correlation. *p*-value reflects the significance level of correlation. *p*-value < 0.05 is represented by*, *p*-value < 0.01 is represented by**, and *p*-value < 0.001 is represented by***. **(D–I)** STRING and Cytoscape were used to visualize the networks of DAMs (red nodes) and DEGs (green nodes). Red indicates positive correlation, and blue indicates negative correlation.

To interpret the potential relationship between the differentially expressed genes and metabolites, the compound reaction network was generated according to the transcriptomic and metabolomic data. The differential genes, metabolites, and pathway analysis are summarized in Table 1. The Venn diagram showed there are 21 KEGG pathways in both transcriptome and metabolome after tadalafil treatment (Figure 7A). The KEGG pathway enrichment analysis presented that DAMs and DEGs involved are associated with pathways. Our results suggested that DEGs and DAMs were primarily involved in pathways relevant to metabolism of alanine, aspartate glutamate, glycine, serine, threonine, and purine and alanine biosynthesis (Figure 7B). In particular, alanine, aspartate, and glutamate metabolism revealed an obvious differentiation in both metabolomic and integrated omics profiles (Figure 7B). The correlation between DEGs and DAMs was presented in the form of a correlation coefficient heatmap (Figure 7C). D-Erythrose 4-phosphate was positively correlated with the expression of ENSG00000143793 (*C1orf35*), ENSG00000197191 (*CYSRT1*), ENSG00000203814 (*H2BC18*), and ENSG00000125148 (*MT2A*) (Figure 7D). N-Acetyl-L-aspartic acid was positively associated with ENSG00000073067 (*CYP2W1*), ENSG00000050555 (*LAMC3*), and ENSG00000188263 (*IL17REL*) (Figure 7E). 2-Oxoadipic acid was positively related with 4 DEGs, including ENSG00000139269 (*INHBE*), ENSG00000023839 (*ABCC2*), and ENSG00000119977 (*TCTN3*) (Figure 7F). Betaine was correlated with the expression of ENSG00000023171 (*GRAMD1B*) (Figure 7G). sn-Glycerol 3-phosphoethanolamine was positively associated with the expression of ENSG00000162572 (*SCNN1D*) (Figure 7H). Succinate, cyclic adenosine diphosphate ribose, D-aspartic acid, taurine, N-acetyl-L-glutamate, and nicotinamide adenine dinucleotide were positively correlated with 2 DEGs, ENSG00000249884 (*NEDF*), and ENSG00000180535 (*BHLHA15*) (Figure 7I). These DAMs and DEGs were almost associated with cancer cell energy supplement.

**TABLE 1:** Functional enrichment analysis for differential genes and metabolites involved in tadalafil treatment on CRC.

Term	Gene	Metabolite
hsa00250 Alanine, aspartate, and glutamate metabolism	*GPT and ASS1*	Succinate, L-glutamine, D-aspartic acid, and N-acetyl-L-aspartic acid
hsa00230 Purine metabolism	*PDE1A, PDE4B, and GDA*	Glutamine and L-2-aminoglutaramic acid
hsa00220 Arginine biosynthesis	*GPT and ASS1*	L-Glutamine and N-acetyl-L-glutamate
hsa00260 Glycine, serine, and threonine metabolism	*SARDH and PGAM1*	Betaine
hsa00920 Sulfur metabolism	*SUOX*	Succinate and taurine
hsa05230 Central carbon metabolism in cancer	*PGAM1*	Succinate and L-glutamine
hsa00430 Taurine and hypotaurine metabolism	*GGT5*	Taurine
hsa00564 Glycerophospholipid metabolism	*DGKH*	sn-Glycerol 3-phosphoethanolamine
hsa04152 AMPK signaling pathway	*PFKFB4*	Nicotinamide adenine dinucleotide (NAD)
hsa00051 Fructose and mannose metabolism	*PFKFB4*	D-Sorbitol
hsa00120 Primary bile acid biosynthesis	*ACOX2*	Taurine
hsa04925 Aldosterone synthesis and secretion	*RKCE*	Nicotinamide adenine dinucleotide (NAD)
hsa00030 Pentose phosphate pathway	*RBKS*	D-Erythrose 4-phosphate
hsa04922 Glucagon signaling pathway	*PGAM1*	Succinate
hsa00910 Nitrogen metabolism	*CA8*	L-Glutamine
hsa00240 Pyrimidine metabolism	*DCTPP1*	L-Glutamine
hsa04024 cAMP signaling pathway	*PDE4B*	Succinate
hsa00360 Phenylalanine metabolism	*TAT*	Succinate
hsa00350 Tyrosine metabolism	*TAT*	Succinate
hsa00400 Phenylalanine, tyrosine, and tryptophan biosynthesis	*TAT*	D-Erythrose 4-phosphate
hsa00270 Cysteine and methionine metabolism	*TAT*	1-Aminocyclopropanecarboxylic acid

### Validation of mRNA Expression Changes

**FIGURE 8:**
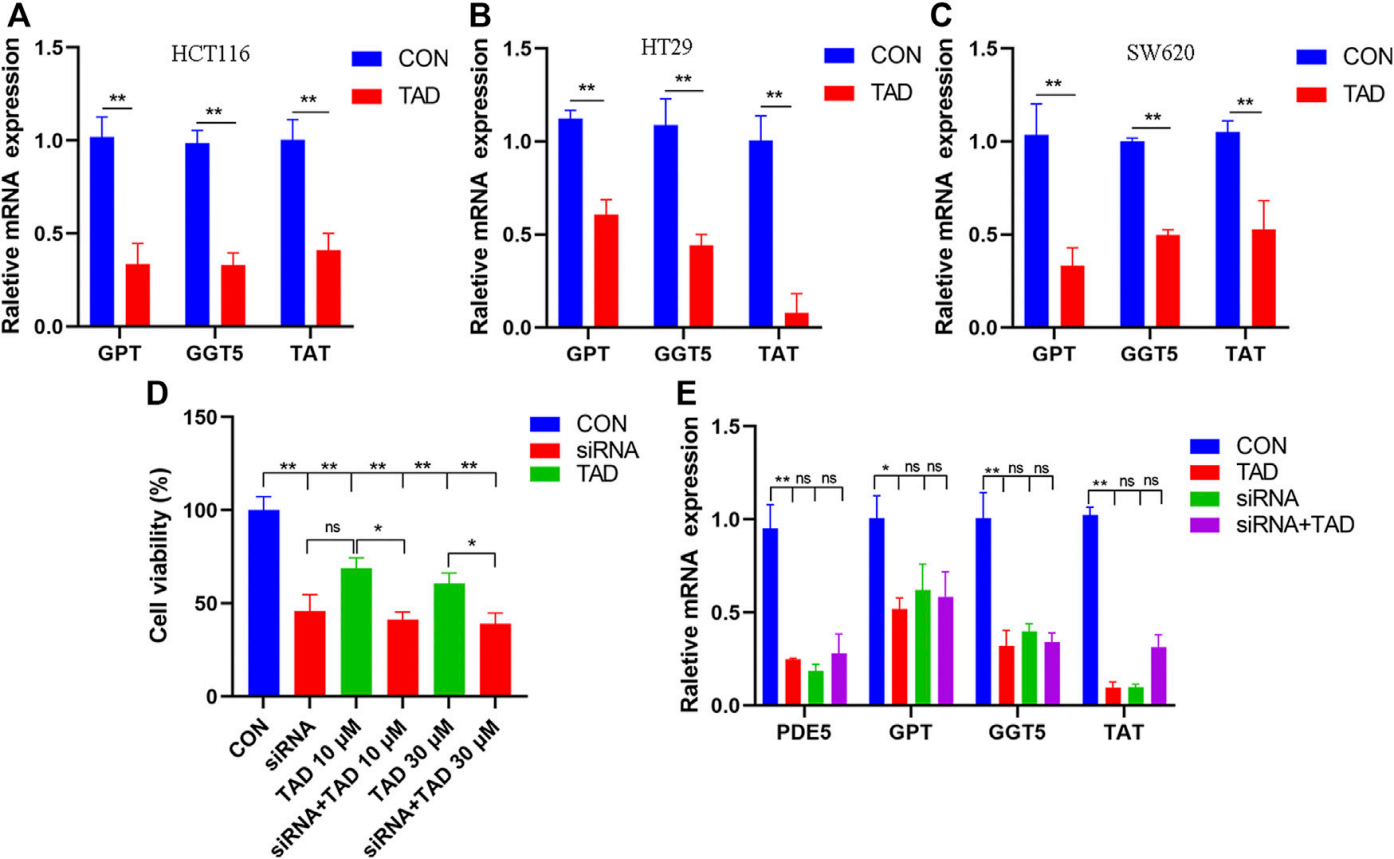
Changes of important genes in amino acid metabolism and target of tadalafil. **(A)** HCT116 cell viability after 10 μM tadalafil treatment for 24 h. **(B)** HT29 cell viability after 10 μM tadalafil treatment for 24 h. **(C)** SW620 cell viability after 10 μM tadalafil treatment for 24 h. **(D)** Cell viability analysis when pde5 was knocked down by siRNA. **(E)** Expression of *GPT, GGT5 and TAT* after tadalafil treatment with or without pde5 knockdown.

The differential expression of amino acid metabolism-related genes, including *GPT, GGT5, and TAT*, was further validated. *GPT*, which encodes glutamate-pyruvate transaminase 1, is an important enzyme which catalyzes the reversible transamination between alanine and 2-oxoglutarate to generate pyruvate and glutamate and therefore plays a key role in the intermediary metabolism of amino acids and glucose. GGT5 is a member of the gamma-glutamyl transpeptidase gene family to participate in the taurine and hypotaurine metabolism by cleaving the gamma-glutamyl moiety of glutathione encode. TAT encodes a mitochondrial protein tyrosine aminotransferase which is related to the level of succinate. We found that the expression of these was all downregulated after tadalafil treatment compared with CON groups in CRC cell lines, HCT116, HT29, and SW620 (Figures 8A–C), and these results were consistent with the transcriptomics analysis. In addition, to explore the anti-tumor pharmacological targets of tadalafil on CRC, we knocked down its canonic target gene *pde5* by small interfering RNA (siRNA) and then analyzed the cell viability after different concentrations of tadalafil treatment with or without pde5 knockdown. The results suggested that tadalafil capacity of anti-tumor partially depended on pde5 (Figure 8D) and its effect on amino acid metabolism also depended on pde5, which was presented by the downregulation of the expression of *GPT, GGT5, and TAT* in tadalafil treatment with pde5 knockdown groups (Figure 8E).

## Discussion

Tadalafil, a PDE5 inhibitor, has been proposed as a potential drug for chemoprevention of CRC. Animal models and population-based studies suggested its potential role in anticancer activity and prolonged survival. Although there were many different anti-tumor mechanisms involved in tadalafil which have been explored, these studies mostly focused on the specific molecular targets in traditional pathways. Therefore, it is hard to thoroughly interpret the pharmacological mechanisms. To further clarify the anti-tumor mechanism, a multi-omics approach was employed to provide insights with respect to the global perturbation of tadalafil treatment, which can provide a systematic perspective of endogenous metabolic changes in response to drug treatment.

In the present study, we observed the changes of gene expression and metabolite after HCT116 was treated with tadalafil by transcriptomics and metabolomics analyses. Alanine, aspartate, and glutamate metabolic pathways were the most obviously changed pathways after tadalafil treatment, in which two genes including *GPT* and *ASS1* and four metabolites including succinate, L-glutamine, D-aspartic acid, and N-acetyl-L-aspartic acid were involved in. Glutamate-pyruvate transaminase 1 is encoded by *GPT* and arginosuccinate synthetase 1 is encoded by ASS1, which play a key role in the intermediary metabolism of amino acids. Numerous studies have confirmed that *ASS1*, considered a house-keeping gene in normal cells and a rate-limiting biosynthetic enzyme for arginine in cells, is absent in many tumors (Dillon et al., 2004; Szlosarek et al., 2007; Delage et al., 2010; Kobayashi et al., 2010). Two studies have indicated that *ASS1* reduced colony forming, proliferation, and invasion of sarcoma and bladder cancer cells and functions as a tumor suppressor gene ((Huang et al., 2013; Allen et al., 2014). Given the importance of arginine in cellular processes of tumors, making the overexpression of *ASS1* in tumor cells suppresses cell growth, and arginine deprivation became a highly promising therapeutic strategy. In this study, the expression of *ASS1* was significantly increased after tadalafil treatment in CRC cell lines, HCT116, HT29, and SW620. This suggested that tadalafil inhibited the proliferation of CRC by decreasing arginine synthesis via improving the expression of *ASS1*.

Succinate, an intermediate of the tricarboxylic acid (TCA) cycle, was one of the metabolites significantly enriched in the alanine, aspartate, and glutamate metabolic pathway. Recently, it has been proved that succinate not only plays a major role in adenosine triphosphate (ATP) generation in mitochondria but also extends into the realms of immunity and cancer (Jiang and Yan, 2017). Succinate apparently decreased in the tadalafil-treated group, indicating that it is another target of inhibition function of tadalafil on CRC. Moreover, L-glutamine, a key intermediate of glutamine metabolism/glutamate, has been proposed as an attractive target for cancer therapy (Korangath et al., 2015). In a word, the anti-tumor effect of tadalafil on CRC is the result of multiple factors including tumor suppressor gene *ASS1* and metabolic intermediates, such as succinate and L-glutamine.

There are some strengths and limitations to the present study when explaining the results. To our knowledge, this study was the first to comprehensively research the mechanism of tadalafil on colorectal cancer cells and found that alanine, aspartate, and glutamate metabolism may be a drug target for anti-tumor activites of tadalafil. We have proved that the effect of tadalafil capacity of anti-tumor on CRC completely depends on PDE5, a classical target of tadalafil, by cell viability and the relative expression of important genes in amino acid metabolism including *GPTA, GGT5*, and TAT in tadalafil with pde5 knockdown groups compared with tadalafil-treated groups. However, the signaling pathway regulated by pde5 was responsible for anti-tumor activity of tadalafil and if there were additional potential targets of tadalafil in CRC remains unclear. Compared to normal colonic mucosa, PDE5 has a high expression in human CRC cell lines and in colon adenomas and adenocarcinomas (Lin et al., 2017), and PDE5 inhibition was found to have anti-tumor activity and potential in the inhibition of CRC (Yarla et al., 2019). The nitric oxide (NO)/cyclic guanosine monophosphate (cGMP) signaling pathway is a major pathway that PDE5 acts principally on, which regulates cell proliferation, tumor development, and tumor progression (Peak et al., 2016).The relationship between the level of cGMP and amino acid metabolism may be a future research direction that clarifies the mechanism of tadalafil treatment in CRC. Moreover, there may be other targets, such as PRMT5, an arginine methyltransferase. Two studies have reported that overexpression of PRMT5 promoted the proliferative, migratory, and colony-forming abilities of CRC cells, but when PRMT5 was knocked down, pharmacological inhibition had the opposite effect. So, PRMT5 has become an increasingly attractive therapeutic target in clinics (Zhang et al., 2015; Prabhu et al., 2017). Our group discovered that tadalafil can affect the methyltransferase activity of PRMT5. In addition, the limited source of CRC cell lines was used in this study. Hence, future research could work on identifying target of tadalafil in regulation of amino acid metabolism and inspecting the generality of this result in different cell lines and different types of CRC.

## Conclusion

Our study demonstrated the anti-tumor effect of tadalafil on colorectal cancer. Integrated transcriptomics and metabolomics revealed that the anti-tumor activity of tadalafil may be related to the amino acid metabolism and purine metabolism. In addition, disturbance of the alanine, aspartate, and glutamate metabolism may account for the main mechanism of tadalafil anti-tumor activity. Altogether, our work provides a new insight into exploring the potential mechanisms of tadalafil, which is significant for pharmacological research and drug repurposing in cancer treatment in clinic.

## Data Availability

The original contributions presented in the study are publicly available. These data can be found at: https://www.ncbi.nlm.nih.gov/bioproject/, PRJNA777785.
