# Supplementary material for: Integration of Transcriptomics and Metabolomics Reveals the Antitumor Mechanism Underlying Tadalafil in Colorectal Cancer
Source: Front Pharmacol. 2022 May 27;13:793499. doi: 10.3389/fphar.2022.793499 (PMC9184725; doi:10.3389/fphar.2022.793499)
Supplement: Supplementary file 1 [file Table1.docx]

**Table S1.** QPCR primers sequence and siRNA oligo primer sequence

| Genes | Forward | Reverse |
| --- | --- | --- |
| **GPT** | GGGTTCGCAGTTCCACTCATT | CCGCACACTCATCAGCTTCA |
| **ASS1** | TCCGTGGTTCTGGCCTACA | GGCTTCCTCGAAGTCTTCCTT |
| **TAT** | TGCCGGGAAAAATGAAAGGC | CTCGGATGGGGTTGAAAGTTT |
| **SARDH** | GGAGGAGACGGGACTACACA | AGCCTCTTGTACTCGTCCAGG |
| **PGAM1** | GTGCAGAAGAGAGCGATCCG | CGGTTAGACCCCCATAGTGC |
| **ACTIN** | CTCCATCCTGGCCTCGCTGT | GCTGTCACCTTCACCGTT CC |
| PDE5-siRNA | Sense | Antisense |
| **siRNA-oligo1** | GCAUAUCCAUGGACUGAUATT | UAUCAGUCCAUGGAUAUGCTT |
| **siRNA-oligo2** | GCUCAGCUCUAUGAGACUUTT | AAGUCUCAUAGAGCUGAGCTT |
